# Quantitative assessment of airborne exposures generated during common cleaning tasks: a pilot study

**DOI:** 10.1186/1476-069X-9-76

**Published:** 2010-11-30

**Authors:** Anila Bello, Margaret M Quinn, Melissa J Perry, Donald K Milton

**Affiliations:** 1 Department of Environmental Health, Harvard School of Public Health, 410 Park Drive, Boston, MA- 02215, USA; 2 Department of Work Environment, University of Massachusetts Lowell, One University Avenue, Lowell, MA- 01854, USA; 3 Maryland Institute for Applied Environmental Health, School of Public Health, University of Maryland, College Park, MD -20742, USA

## Abstract

**Background:**

A growing body of epidemiologic evidence suggests an association between exposure to cleaning products with asthma and other respiratory disorders. Thus far, these studies have conducted only limited quantitative exposure assessments. Exposures from cleaning products are difficult to measure because they are complex mixtures of chemicals with a range of physicochemical properties, thus requiring multiple measurement techniques. We conducted a pilot exposure assessment study to identify methods for assessing short term, task-based airborne exposures and to quantitatively evaluate airborne exposures associated with cleaning tasks simulated under controlled work environment conditions.

**Methods:**

Sink, mirror, and toilet bowl cleaning tasks were simulated in a large ventilated bathroom and a small unventilated bathroom using a general purpose, a glass, and a bathroom cleaner. All tasks were performed for 10 minutes. Airborne total volatile organic compounds (TVOC) generated during the tasks were measured using a direct reading instrument (DRI) with a photo ionization detector. Volatile organic ingredients of the cleaning mixtures were assessed utilizing an integrated sampling and analytic method, EPA TO-17. Ammonia air concentrations were also measured with an electrochemical sensor embedded in the DRI.

**Results:**

Average TVOC concentrations calculated for 10 minute tasks ranged 0.02 - 6.49 ppm and the highest peak concentrations observed ranged 0.14-11 ppm. TVOC time concentration profiles indicated that exposures above background level remained present for about 20 minutes after cessation of the tasks. Among several targeted VOC compounds from cleaning mixtures, only 2-BE was detectable with the EPA method. The ten minute average 2- BE concentrations ranged 0.30 -21 ppm between tasks. The DRI underestimated 2-BE exposures compared to the results from the integrated method. The highest concentration of ammonia of 2.8 ppm occurred during mirror cleaning.

**Conclusions:**

Our results indicate that airborne exposures from short-term cleaning tasks can remain in the air even after tasks' cessation, suggesting potential exposures to anyone entering the room shortly after cleaning. Additionally, 2-BE concentrations from cleaning could approach occupational exposure limits and warrant further investigation. Measurement methods applied in this study can be useful for workplace assessment of airborne exposures during cleaning, if the limitations identified here are addressed.

## Background

A growing body of epidemiologic evidence suggests that workers who perform institutional and domestic cleaning are at increased risks for asthma and other respiratory diseases [1-14]. Very few studies to date have carried out quantitative assessment of workplace cleaning exposures [15-18]. Often qualitative exposure data, such as job titles and product types are used to represent exposure in epidemiologic investigations of asthma from cleaning. Quantitative exposure assessments are necessary for investigations of ingredients potentially responsible for respiratory symptoms among cleaning workers and to evaluate exposure-response relationships [19]. A recent review of asthma and cleaning by Zock et al. [20] emphasized the need for quantitative exposure assessment studies.

Airborne exposures from cleaning products are challenging to quantity because they are complex mixtures of ingredients having a range of volatilities and other physicochemical properties and thus require multiple measurement techniques [21,22]. An additional challenge for exposure studies is to identify methods that can measure short-term and peak exposures, which are important determinants of respiratory symptoms [23,24].

The type and the frequency of products used depend on the cleaning task. Multiple cleaning tasks may be performed in one room and, for cleaners in institutions like hospitals and schools, the set of cleaning tasks may be performed repeatedly during the day [10,21]. We therefore designed a task-based assessment that can provide better evaluation of exposure variability, instead of assessing personal exposures using continuous 8-hour time weighted average measurements. Additionally, by using the task as the unit of analysis, one can investigate short term or peak exposures, as determinants of respiratory symptoms. Finally, the results of task based assessments can assist in the development of questionnaires for estimating cleaning workers' exposures when measurements are not available.

We conducted a task-based exposure assessment study with two main objectives: a) to identify methods for assessing short term, task-based airborne exposures; and b) to evaluate the airborne exposures associated with cleaning tasks simulated under controlled work environment conditions. Results of this work can provide a foundation for developing a quantitative workplace exposure assessment strategy for an epidemiologic investigation.

## Methods

### Selection of cleaning products

In an earlier study, we identified cleaning products used for common cleaning activities in six hospitals in Massachusetts. Detailed information on products identified and their chemical compositions are described elsewhere [21]. A set of frequently used products was selected for further quantitative exposure characterization, including a glass cleaner, a general purpose cleaner, and a bathroom cleaner (Table 1). Selection criteria specified that the product must: 1) contain at least one volatile ingredient identified as a potential respiratory hazard based on our previous qualitative assessment [21]; 2) be task specific; 3) be available via commonly used distributers. Material Safety Data Sheets (MSDSs) indicated 2-buthoxyethanol (2-BE) was a major ingredient in all of the products selected, with concentrations ranging from 0.5% - 10% by weight in the bulk products (see Table 1). Other volatile ingredients listed on the MSDS for these mixtures were ethanolamine, ethylene glycol, ethanol, and propylene glycol monoethyl ether.

**Table 1:** Ingredients of cleaning products used for simulation of cleaning tasks

Product	Material Safety Data Sheets' (MSDS) ingredients	CAS number	% by weight
**Glass cleaner**		111-76-2	25-40
*1) concentrate*	2- Butoxyethanol	107-98-2	5-7
	Propylene glycol monomethyl ether	NA	5-7
	Alcohol ethoxy sulfate	1336-21-6	3-5
	Ammonium hydroxide	64-02-8	1-3
	Tetrasodium ethylenediamine tetraacetate	64-17-5	0.25-1.0
	Ethyl alcohol		
			
*2) ready to use*	Ammonium Hydroxide	1336-21-6	3-5

**General Purpose cleaner**			
*1) concentrate*	2-Buthoxyethanol	111-76-2	35-45
	Ethanolamine	141-43-5	10-20
	Sodium hydroxide	1310-73-2	1-1.5
			
*2) ready to use*	Mono-ethanolamine	141-43-5	1-3
	2-Buthoxyethanol	111-76-2	5-7

**Bathroom cleaner**			
*1) concentrate*	2-Buthoxyethanol	111-76-2	25-40
	Secondary alcohol ethoxylate	68131-40-8	10-25
	Ethanolamine	141-43-5	7-10
	Fragrance	NA	3-5
	Tetrasodium ethylenediamine tetraacetate	64-02-8	1-1.5
	N-Alkyl dimethyl benzyl ammonium chloride	68-424-85-1	0.25-1
	Didecyl dimethyl ammonium chloride	7173-51-5	< 0.1
			
*2) ready to use*	2-Buthoxyethanol	111-76-2	1-3

### Simulations of cleaning tasks

Worksite observational and video analyses of cleaning tasks in two hospitals and one university in Massachusetts were conducted. These analyses focused on bathrooms which our previous qualitative assessment recognized as requiring multiple cleaning tasks. We identified workplace practices related to product application methods, worker's physical movements and proximity to cleaning products, average task duration, and typical room dimensions in which the tasks were performed. The findings from these worksite analyses then were used to develop simulations of the cleaning tasks.

Using the products selected, we simulated three types of cleaning tasks: mirror cleaning (with the glass cleaner), sink cleaning (with the general purpose cleaner) and toilet bowl cleaning (with the bathroom cleaner). Products were sprayed and then wiped using paper towels for mirror and sink cleaning; and a brush for toilet bowl cleaning, as commonly done at the worksite.

The main reason for performing simulations was to control task frequency, duration, and environmental conditions such as ventilation and possible interferences from other sources of volatile compounds. Pilot simulations were initially performed to determine the duration of cleaning tasks needed to collect a sufficient amount of analyte to reach the limit of detection (LOD) of the analytical method, while aiming to conduct the tasks within their actual workplace durations. Workplace observations showed that durations between the tasks varied from 3-10 minutes, depending on the surface dirtiness and the number of toilet bowls, sink or mirrors in one bathroom. After several simulations and measurements with methods described below, the final task duration was determined as 10 minutes. Integrated air sampling was conducted for each task for the entire simulation period. Direct reading measurements were performed at the same time, but also continued after the tasks stopped, in order to evaluate the after -task exposure profiles.

**Figure 1:**
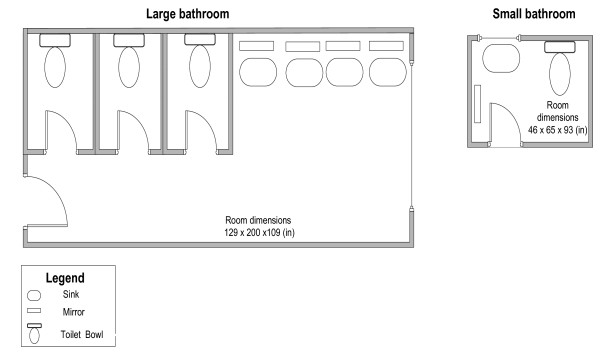
**Schematic presentation of the bathrooms where cleaning tasks were performed**.

To investigate the feasibility of capturing a wide range of airborne concentrations (representing lower and higher exposures), cleaning tasks were simulated with varying conditions: in a small and large bathroom; with or without ventilation; and with products at different dilution concentrations. It was hypothesized that factors such as the volume of the room, ventilation conditions, concentrations of the volatile ingredient in the products, and amount of the product used per task, would be important exposure determinants. The small bathroom had dimensions typical of a single patient hospital bathroom and the large bathroom had dimensions typical of a public bathroom with three toilet stalls, four sinks and mirrors (Figure 1). The large bathroom was continuously ventilated with an air exchange rate of 5.5 air changes/hour. The small bathroom's ventilation was controlled using the exhaust fan, which was turned off during the simulations. Amount of the product consumed during each task was recorded by weighing the product bottle before and after each task. The doors and windows were kept closed during the simulations and were opened only after cleaning tasks and measurement had stopped. Paper towels used were removed from the bathrooms after cleaning.

### Airborne measurement methods

Volatile organic compounds were assessed using the following metrics: 1) total volatile organic compounds (TVOC) with direct reading measurements methods; 2) volatile organic compounds (VOC) with a standard integrated sampling and analytical method. Ammonia, a specific ingredient with known respiratory effects, was also assessed concurrently with other VOC metrics. The following measurement methods were used during the simulations:

Direct reading measurements of TVOC: Concentrations of TVOC in air were measured using a direct reading instrument (DRI) with a photo ionization detector (PID), Gray Wolf Sensing Solution, the Direct Sense TVOC-TG-502, Trumbull, CT. The PID was equipped with a parts per billion (ppb) sensor with a measurement range of 0.02 -20 parts per million (ppm). Calibration of the ppb sensor was performed at two calibration points: 0 ppm using free air and 7.5 ppm using isobutylene. Concentrations of TVOC were recorded every 15 seconds using a pocket personal computer (PC) connected to the air sampling probe. The data were processed with the Active Sync Software 4.2 and Gray Wolf software version 2.12. The instrument was held constantly in the breathing zone of the person who performed the tasks. Background concentrations of TVOC in the bathrooms were measured before each task. TVOC concentration profiles were obtained during the 10 minutes of the cleaning tasks and continued after their cessation, until the TVOC concentrations dropped to the background level.

Integrated sampling and analytical method, EPA TO -17: Integrated sampling was conducted simultaneously with the direct reading TVOC measurements. Breathing zone samples were collected in duplicates on the person who performed the tasks. Active sampling was conducted using the Perkins-Elmer ATD 400 thermal desorption tubes at a flow rate of 65- 70 ml/min. Samples were collected continuously for the 10 minutes of the tasks. Following sampling, the tubes were refrigerated and later transported in ice bags for chemical analysis. Compounds sampled were recovered with thermal desorption and analyzed with an Agilent 6890/5973 GC/MS with analytical column J&W DB-1, using helium as the carrier gas.

Ammonia measures: Ammonia was measured with an electrochemical sensor, which was embedded in the DRI. Similar to TVOC, ammonia concentration-time profiles were obtained during and after each task. The data were recorded and downloaded simultaneously with TVOC using the same software.

## Results

**Figure 2:**
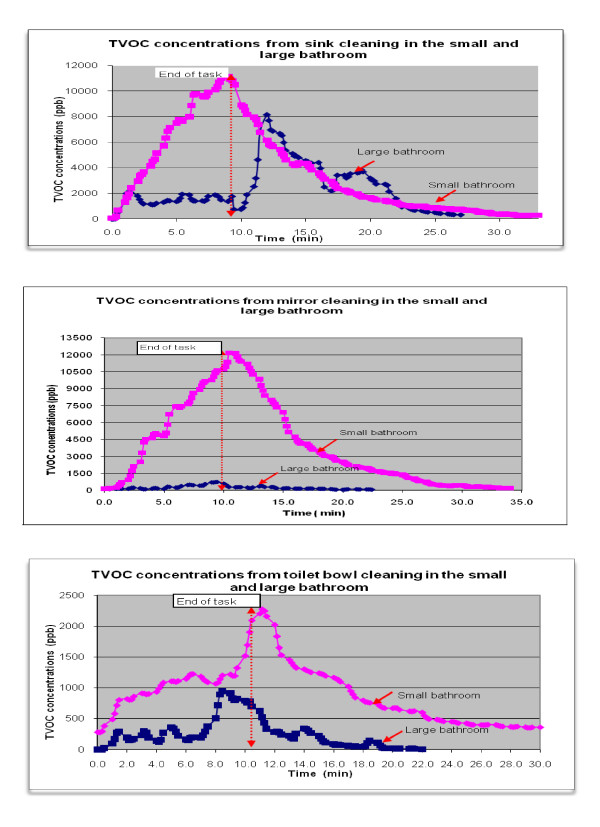
**Profile of Total Volatile Organic Compounds (TVOC) concentrations during and after cleaning tasks (task duration 10 minutes)**.

TVOC concentrations: Real time concentration profiles of TVOC for sink, mirror, and toilet bowl cleaning tasks (Figure 2) show TVOC concentrations steadily increasing with time during task performance, reaching the peak at the end of the cleaning period. TVOC concentrations after the tasks declined exponentially to background concentrations. The time to reach the background level typically was about 20 minutes after the tasks had stopped.

Average TVOC concentrations calculated for 10 minute tasks ranged from 0.02 - 6.49 ppm and the highest peak concentrations observed for each task ranged from 0.14-11 ppm (Table 2). Overall, concentrations varied by task type, room size, ventilation status, and dilution rate of the product used. Amount of products used did not change much between tasks. The highest peak concentrations were detected during sink and mirror cleaning in the small bathroom without ventilation. Average and peak concentrations were higher in the small bathroom than in the larger one. TVOC profiles show a steady concentration increase in the small bathroom, while in the large bathroom the values were lower and tended to fluctuate more. Variability of TVOC concentrations in the large bathroom can be related to the air mixing from ventilation and movement of the person performing the task from one sink to another. As expected, we found that airborne concentrations were higher when the more concentrated products were used.

**Table 2:** Concentrations of 2-butoxyethanol (2-BE) and Total Volatile Organic Compounds (TVOC) measured simultaneously during 10 minutes tasks

Environment/Task	Product type	Product dilution status	**Conc. of 2-BE in the product **(% by weight)	**Average 2-BE air concentrations in ppm (sd)**^**B**^	**TVOC average concentrations ppm (sd)**^**C**^	**TVOC peak concentrations ppm **^**D**^
**Small Bathroom **^**A**^						

Sink cleaning	General purpose	Ready to Use (RTU)	5-7	21.27 (2.96)	6.49 (3.56)	11.11

Sink cleaning	General purpose	1 part RTU: 1 water	2.5-3.5	13.32 (2.54)	2.54 (1.51)	4.31

Mirror cleaning	Glass cleaner	1 part concentrated form: 4 parts of water	6-10	13.08 (1.45)	5.26 (3.54)	11.36

Mirror cleaning	Glass cleaner	1 part conc .form: 19 parts of water	1-2	2.96 (0.23)	0.74 (0.39)	1.46

Toilet bowl cleaning	Bathroom cleaner	Ready to Use (RTU)	1-3	3.74 (0.36)	0.96 (0.28)	2.2

Toilet bowl cleaning	Bathroom cleaner	1 part RTU: 1 water	0.5-1.5	2.70 (0.34)	0.56 (0.16)	0.71

**Large Bathroom**						

Sink cleaning	General purpose	Ready to Use (RTU)	5-7	6.27 (0)	1.36 (0.51)	2.13

Sink cleaning	General purpose	1 part RTU: 1 water	2.5-3.5	3.30 (0.1)	0.61 (0.30)	1.37

Mirror cleaning	Glass cleaner	1 part concentrated form: 4 parts of water	6-10	1.98 (0.12)	0.30 (0.19)	0.74

Mirror cleaning	Glass cleaner	1 part conc .form: 19 parts of water	1-2	0.32 (0.01)	0.02 (0.03)	0.14

Toilet bowl cleaning	Bathroom cleaner	Ready to Use (RTU)	1-3	3.05 (0.81)	0.32 (0.27)	0.95

Toilet bowl cleaning	Bathroom cleaner	1 part RTU: 1 water	0.5-1.5	2.76 (0.45)	0.11 (0.08)	0.29

^A) ^Tasks were performed without using the bathroom fan

^B) ^Each value is the average of two side-by side breathing zone samples collected and analyzed according integrated sampling method EPA TO-17

^C) ^The 10 minute average TVOC concentrations measured using a photo-ionization detector (PID)

^D) ^The highest TVOC concentrations recorded by the PID during 10 min tasks.

**Figure 3:**
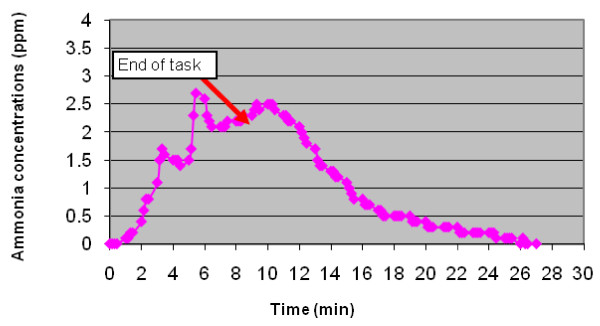
**Ammonia concentrations profile during and after mirror cleaning in the small bathroom**.

Concentrations of ammonia: The highest peak concentration of ammonia (2.8 ppm) was detected during mirror cleaning (Figure 3), when using the concentrated product that contained 3-5% by weight ammonium hydroxide. Concentration time profiles indicated that ammonia was present even after the tasks had stopped. Lower concentrations were recorded during toilet bowl cleaning (peak of 0.2 ppm), from the product that contained quaternary ammonium compounds at < 1.5% in the concentrated form.

Concentrations of 2-Buthoxyethanol: 2- BE was the only VOC measured by the EPA TO- 17 for the 10 minute sampling. Other target VOC compounds were not detectable from the samples collected. Average concentrations of 2-BE generated from different tasks ranged from 0.3-21 ppm. Airborne concentrations were higher: when products with higher percentage of 2-BE were used; during sink and mirror cleaning compared to toilet bowl cleaning; and when tasks were performed in the small bathroom compared to the large one. The highest concentrations were measured during sink cleaning, when the general purpose cleaner containing 5 - 7% 2-BE was used.

Correlation of TVOC with 2-BE: Contrary to expectations, the TVOC measurements were consistently lower than 2-BE, a single volatile organic compound of the mixture (Table 2). However, good correlation was found between TVOC and 2-BE values measured simultaneously for the same task (R^2 ^= 0.94).

## Discussion

In this study we assessed quantitatively airborne exposures generated from cleaning tasks performed under controlled work environment conditions. Several exposure measures such as TVOC, 2-butoxyethanol (2-BE) and ammonia were assessed with selected measurement methods. Our results show that VOC exposures remain airborne even after the cessation of cleaning tasks, suggesting potential exposure to anyone entering the room shortly after cleaning. Additionally, the results indicated that 2-BE peak concentrations from cleaning can approach occupational exposure limits, warranting further workplace investigations. The quantitative exposure measurements reported here contribute to the limited workplace exposure data in the literature related to cleaning. The main conclusions are discussed below:

1) The measurement methods we applied for assessing exposures from cleaning tasks are useful for future studies, with limitations.

We utilized several measurement methods for quantifying different exposure metrics including integrated sampling and analytical method of EPA TO-17. This method was selected because it provides detection of VOC at low concentrations and grants collection of compounds with a wide range of volatilities (e.g. ethanol BP = 78°C; 2-BE BP = 218 °C) by utilizing multi-media sorbent sampling tubes. However, for the 10 minute sampling only 2-BE was detectable with the method. The initial list of target volatile ingredients from the MSDS data included ethanol, 1-methoxy 2- propanol, and ethanolamine. Ethanolamine was removed from the target list because it was not amenable to the method. Ethanol &1-methoxy 2- propanol concentrations were lower than the LOD, for the 10 minute sampling period. For longer sampling periods (such as 20 minutes, data not shown) ethanol, 1-methoxy 2- propane were detectable with the EPA method. Given that our goal of capturing a range of VOC with this method was not achieved (either because of short term sampling or low product concentrations of target compounds), specific measurement of individual compounds may be more feasible to apply for workplace exposure assessment. For example, 2- BE can be measured using the NIOSH 1430 method.

TVOC measured with the DRI- PID underestimated exposures from cleaning activities. Given that the TVOC metric represents the sum of the volatile compounds of the mixture, including 2-BE, one would expect that the value of TVOC would be higher than the single ingredient. Because the 2-BE ionization potential (IP) is lower than the IP of the PID lamp (IP for 2-BE is 8.6 eV vs. 10.6 eV), we expected that 2-BE would be measured by the PID. However, our data indicate a clear 2-BE exposure underestimation by the PID. A possible explanation may be related to the differences in sampling methods between integrated sampling, which is based on active sampling; and the real time sampling, which is based on diffusion. Aerosol particles generated during product spraying may be captured by active sampling and not by the PID, therefore producing higher 2-BE levels by integrated sampling.

The same underestimation of VOC from the PID was observed by Coy et al. [25]. This study compared PID results with integrated sampling during simultaneous measurements from the same solvent mixtures. The authors suggest that PID response underestimation is related to: a) different ionization potential of the individual compounds of the mixture; b) non-linearity of the PID response for high concentrations (2000 ppm); c) the size of the ionization chamber. Consistent with our findings, this study showed high correlation of the PID response with integrated sampling measures.

Due to the observed underestimation, we recommend that TVOC -DRI measurement for cleaning mixtures be conducted only when the limitations are taken into account. DRI can be used for: a) initial screening of TVOC concentrations; b) for evaluating exposure control strategies; and c) for identifying exposure peaks and exposure dynamics, which can be useful for prioritizing activities for further and more precise quantitative measures.

2) Quantification of airborne exposures from cleaning requires investigation of other exposure metrics and a variety of sampling and analytical techniques.

In addition to the exposure metrics considered here (TVOC, 2-BE, ammonia) other metrics can be considered for a comprehensive quantitative exposure assessment strategy. These include assessments of additional chemical agents with important health relevance for respiratory irritation and sensitization, such as quaternary ammonium compounds (quats), ethanolamines, and phenols. Quantitative assessment of these ingredients cannot be achieved with one single method, given that these chemicals have different chemical and physical properties. For example quats can be measured with Ion Chromatography [26] and ethanolamines can be measured with GC/FID NIOSH 2007 method.

Further considerations for quantitative assessment can include aerosol exposure characterizations. Product spraying, a common activity during cleaning, generates liquid aerosols of variable chemical composition, including non-volatile compounds such as quats that have been associated with asthma symptoms in several case reports [27]. However, current literature lacks the evidence on size distributions of particles generated from spraying during cleaning. Determination of respirable or ultrafine particle concentrations from spraying may provide a better understanding of cleaning related health effects. Additionally, there are no studies to date that focus on assessment of aerosol dust particles present in indoor environments as potential carriers of volatile and nonvolatile ingredients from cleaning. Secondary emissions generated from cleaning chemicals reaction with ozone, which have been investigated by experimental studies [17], may also be important to consider when developing quantitative workplace exposure assessment strategies.

3) The quantitative findings for airborne TVOC and 2-BE suggest that common cleaning tasks contribute to poor indoor quality and may present a risk of adverse health effects.

The highest TVOC peak concentrations (approximately 11 ppm) and 10 minute average concentrations (approximately 6 ppm) were measured when the general purpose cleaner was used in the small unventilated bathroom. Although occupational and environmental standards for indoor air TVOC have not been established, Molhave et al. [28,29] proposed indoor TVOC concentrations of increasing concern for health effects as follows: a comfort range (< 0.2 mg/m^3 ^), a multi factorial exposure range (0.2-3 mg/m^3 ^); a discomfort range (3- 25 mg/m^3 ^); and a toxic range (> 25 mg/m^3^). Our peak TVOC concentration data converted to mg/m^3 ^(isobutylene equivalent) ranged from 0.66-26 mg/m^3^. Concentrations we recorded for most of the tasks fall into the discomfort range. These results suggest that cleaning can make a significant contribution to the poor indoor air quality. Additionally, Molhave et al. [29] recommended that if a direct reading detector indicates concentrations above 0.3 mg/m^3 ^, further detailed exposure assessments for health effects evaluations are essential.

TVOC concentrations have been measured in several indoor environments including offices, schools, homes, and hospitals [29-34]. In these settings, the TVOC ranged from 1-25 mg/m^3 ^and were expressed as the average values for different time durations, from hours to days of air sampling. Even though these studies recognize the possibility of higher short-term TVOC exposures, peak TVOC- activity specific data which are important for asthma assessment, have not been evaluated [23]. Because the degree of cleaning contribution to the short term peak exposures is unknown, further assessments in the workplace are needed.

Airborne concentrations from cleaning 2-BE may be a concern in the workplace. Concentrations of 2-BE measured here ranged widely among the tasks, with the highest values obtained when the general purpose cleaner with 5-7% 2-BE by weight was used in the small bathroom, approximately 21 ppm. California Proposition 65 has set the Reference Exposure Limit (REL) for 2-BE at 2.9 ppm for one hour of exposure. Our 2-BE results suggest that application of a general purpose cleaner continuously for several consecutive tasks in the workplace can easily result in worker's exposure higher than the California REL limit.

Several laboratory emissions studies have measured 2-BE concentrations from cleaning products. Slightly lower air concentrations than ours were reported by Zhu et al. [35] in an experimental study that determined 2-BE emission factors using a field and laboratory emission cell (FLEC). One hour concentrations of 2-BE ranged from 2.8-62 mg/m^3 ^(0.57- 12.6 ppm). Singer et al. [18] investigated emission profiles of 2-BE from several cleaning products and reported concentrations of 0. 33-2.3 mg/m^3 ^over one hour of exposure.

There are very limited workplace exposure data of 2-BE from cleaning. Occupational standards for 2-BE such as OSHA Permissible Exposure Limit (PEL) of 8 hr TWA is 50 ppm and NIOSH Recommended Exposure Limit (REL) for 10 hr TWA is 5 ppm (24 mg/m^3^). Vincent and coworkers in 1993 [16] assessed 2-BE workplace exposures for 29 cleaning workers, which ranged from 0.1-7.33 ppm for 8 hour TWA. 2-BE exposures from cleaning may meet OSHA regulations, however, compliance does not always imply that workers are protected from respiratory irritation symptoms from short term peak exposures [23,36]. Our findings of concentrations as high as 21 ppm, although not directly comparable with the occupational standards, warrant further assessment of 2-BE from cleaning in the workplace.

Ammonia concentrations from the tasks performed (0.01-2.8 ppm) were low compared to OSHA -PEL 8 hour TWA of 50 ppm and NIOSH short term exposure limit (STEL) 15- min TWA of 35 ppm. Several studies have associated inorganic gases such as ammonia and chlorine with irritation symptoms reported among cleaning workers [17,15]. Concentrations of ammonia reported by Ramon and coworkers range from 0.6-6.4 ppm with peaks over 50 ppm during domestic cleaning tasks [15]. Lower concentrations were reported by Fedoruk et al. [37] when assessing airborne ammonia from a window and a bathroom tile cleaner. This study concluded that standard cleaning solutions are unlikely to produce significant ammonia exposures, but the authors advise that application of more concentrated products (e.g. > 3%) in poorly ventilated areas may be of concern.

4) Concentrations of TVOC measured after cleaning suggests that exposures may affect not only workers involved in cleaning but also other building occupants.

Real time TVOC concentration profiles after the cessation of cleaning tasks indicated that it takes more than 20 minutes after cleaning for exposures to decline to background levels. This finding relates to a single application of one product used during one task, especially in the small unventilated room. It would be expected that multiple tasks performed consecutively would generate higher exposure concentrations requiring longer decay times. These results suggest that not only workers involved with cleaning, but others, who are present in the room after cleaning, are potentially exposed. Several emissions studies conducted in laboratory chambers have suggested that ingredients in cleaning products such as glycol ethers are slowly released in the air even hours after product applications [17,18]. These experimental results indicate that there is a potential risk for exposure to other building occupants not involved with cleaning. Further quantitative investigation in real world scenarios is critical to evaluate airborne exposures after cleaning.

## Conclusions

Measurement methods reported here can be used for workplace assessments of airborne exposures generated during cleaning tasks, if the limitations are addressed. Combinations of individual measurements methods for ingredients of significant health relevance with TVOC direct reading measurements can provide complimentary evidence for an epidemiologic investigation and for developing workplace controls. Additional exposure metrics quantified using a variety of sampling and analytic methods will be needed for more comprehensive quantitative exposure assessment.

Our work also shows that airborne VOC exposures occur during short-term cleaning tasks and that these can remain in the air after the task stops, suggesting potential exposure to anyone entering the room shortly after cleaning. In addition, 2-BE peak concentrations from cleaning could approach occupational exposure limits and warrant further investigation. We recognize that cleaning tasks performed at actual worksites are likely to differ from our simulated tasks in several ways: 1) the duration of tasks is more variable; 2) tasks are performed consecutively in one room (e.g. mirror, sink, and toilet all in one bathroom); and 3) the cleaning task cycle is repeated multiple times in institutions such as hospitals and schools where numerous bathrooms are cleaned in a single day. Due to these differences, workplace exposure concentrations are likely to be different than the values reported here, however these data and the methods used to obtain them can be used as groundwork for conducting a comprehensive quantitative exposure assessment for an epidemiologic investigation.

## List of abbreviations

2-BE: 2-Butoxyethanol; MSDSs: Materials Safety Data Sheets; DRI: Direct Reading Instrument; EPA: Environmental Protection Agency; NIOSH: National Institute for Occupational Health and Safety; OSHA: Occupational Health and Safety Administration; PEL: Permissible Exposure Levels; PID: Photo Ionization Detector; REL: Recommended Exposure Limit; TLV: Threshold Limit Values; TVOC: Total Volatile Organic Compounds; TWA: Time Weighted Average; VOC: Volatile Organic Compounds;

## Competing interests

The authors declare that they have no competing interests.

## Authors' contributions

AB led the design of the research, drafted the paper, carried out simulations, sampling and measurement. MQco-led the design of the research guided paper writing, facilitated workplace site visits and conducted paper revisions. MP and DM provided critical input on simulations design, VOC measurements methods, and paper revisions. All authors approved the final manuscript.
